# Culturally sensitive neonatal palliative care: a critical review

**DOI:** 10.1177/26323524231222499

**Published:** 2024-01-08

**Authors:** Hayley Redman, Marie Clancy, Felicity Thomas

**Affiliations:** Wellcome Centre for Cultures and Environments of Health, University of Exeter, The Queens Drive, Exeter EX4 4QJ, UK; Academy of Nursing, University of Exeter, Exeter, UK; Department of Health and Community Sciences, Wellcome Centre for Cultures and Environments of Health, University of Exeter, Exeter, UK

**Keywords:** cultural diversity, inequalities, neonatal, palliative care, perinatal

## Abstract

Although there are known disparities in neonatal and perinatal deaths across cultural groups, less is known about how cultural diversity impacts neonatal palliative care. This article critically reviews available literature and sets out key questions that need to be addressed to enhance neonatal palliative care provision for culturally diverse families. We begin by critically reviewing the challenges to recording, categorizing and understanding data which need to be addressed to enable a true reflection of the health disparities in neonatal mortality. We then consider whose voices frame the current neonatal palliative care agenda, and, importantly, whose perspectives are missing; what this means in terms of limiting current understanding and how the inclusion of diverse perspectives can potentially help address current inequities in service provision. Utilizing these insights, we make recommendations towards setting a research agenda, including key areas for future enquiry and methodological and practice-based considerations.

## Introduction

The majority of child deaths occur within the first 28 days of life: the neonatal period.^1^ Advances in antenatal screening and diagnostic technologies have improved the identification of conditions which may shorten the lives of unborn babies. These babies, alongside babies diagnosed with a life-limiting condition at or after birth, and their families may require a palliative approach to care.^2,3^

Neonatal palliative care utilizes a holistic approach embracing physical, emotional, social and spiritual elements of care, within a family-centred philosophy.^4,5^ It is an emerging discipline, which emphasizes the comfort of the baby and quality of life for the family, however long their time together may be. It aims to improve the experience and outcomes for families during a time of crisis, specifically when their baby is not expected to survive, or when a baby has multiple complex health needs with an uncertain future.^6^ It often involves complex medical and nursing interventions as well as challenging ethical decision-making, particularly regarding the withholding or withdrawal of life-sustaining treatment. Although often used interchangeably, palliative and end-of-life care are not synonymous – palliative care can include end-of-life care but is much broader and can last for months or years.^7^ Most babies with potential palliative care needs will survive to discharge from the neonatal unit.^8^ As such a very finely balanced consideration of complex risks and long-term implications is needed by clinicians and families particularly during neonatal unit stays.^9^

Guidelines on neonatal and perinatal palliative care in the United Kingdom have been issued by the British Association of Perinatal Medicine (2010), the Royal College of Paediatrics and Child Health (2014), the Royal College of Nursing (2018) and the General Medical Council (2022).^10–13^ Despite this range of different guidelines on neonatal palliative care in place across the United Kingdom, there are inconsistencies in how infants receiving this care are identified, which families receive it and to what extent it differs from routine care.^14^ Moreover, of the 5914 deaths prior to neonatal discharge in England and Wales between 2015 and 2020, 50.7% (3000) did not meet the palliative care criteria identified by the British Association of Perinatal Medicine (BAPM) guidelines.^8^ This is especially concerning when data show marked disparities in neonatal deaths across population groups. Whilst death rates have steadily decreased over the past 5 years, they remain exceptionally high for babies of Black and Black British ethnicity, where stillbirth rates are over twice those for babies of White ethnicity and neonatal mortality rates are 43% higher. Similarly, mortality rates remain high for babies of Asian and Asian British ethnicity with stillbirth and neonatal mortality rates both around 60% higher than for babies of White ethnicity.^15^ Economic factors also play an intersecting role, with data showing that more babies born to mothers living in the most deprived areas of the United Kingdom died in the neonatal period when compared to mothers who lived in less deprived areas.^16,17^

When faced with the need for neonatal palliative care, families are thrust into a world of uncertainty, loss and ethical decision-making complexity. Whilst the significance and impact of providing compassionate care to ethnically diverse families have been noted,^18^ there remains a distinct lack of evidence base to guide neonatal palliative care and the challenging and complex landscape faced by ethnically diverse families and those with a remit to support them.

Maternal health is an important dimension to consider during this uncertain time.^19^ Increasingly, evidence suggests that mothers of children with life-limiting conditions experience higher incidences of serious physical and mental health needs.^20–22^ Recently, high profile reports on systemic and cultural failings in nursing and midwifery (e.g. the Francis Report, 2013; the Ockenden Report, 2022^23,24^) have highlighted the need for concerted, multidisciplinary action to make services safer, more sensitive, and more compassionate. Reports of disparities surrounding pregnancy outcomes of Black and Asian women are not new, dating as far back as the early 1990s. However public outcry has become increasingly audible since the publication of the *Mothers and Babies: Reducing Risk through Audits and Confidential Enquiries Across the UK* report from MBRRACE-UK in 2018.^25^ There is now a significant, and welcome, national drive to reduce the disproportionately high maternal mortality rate among Asian and Black women and this accelerated in response to COVID-19, with reports on how structural and cultural racism adversely affect health.^26,27^ Such findings echo an emerging body of work in migration studies identifying the need for healthcare services to adapt to an increasingly diverse population through embedding cultural competence and cultural humility within healthcare delivery.^28,29^ Yet, while these kinds of interventions aim to improve outcomes before, during and shortly after birth for babies, there are no corresponding strategies or targeted programmes to identify and tackle the impact of health inequalities within neonatal units.^30^

Against this backdrop, this article reviews and sets out key questions that need to be asked to deepen the understanding of neonatal palliative care for culturally diverse families. We chose not to undertake a systematic or scoping review because we wanted to bring together a wide range of resources to identify gaps in current knowledge to identify areas for further research moving forward. Systematic reviews tend to reinforce privilege and silence the experiences of marginalized groups – given our particular focus on identifying the voices missing in the current evidence base a critical review of the literature was deemed more appropriate.

The article will begin by critically reviewing the categorization and recording of ethnicity data collection, and the significant gaps in the data which need to be addressed to understand the true picture of health disparities in neonatal mortality. The second section will consider whose voices frame the current neonatal palliative care agenda, and, importantly, whose perspectives are missing. In setting a research agenda, the final section will consider key areas for further research, including: the lived experiences of families and how they can navigate and negotiate challenges in neonatal palliative care settings; effective communication between families and professionals; the need to provide culturally competent forms of bereavement care for culturally diverse families and the methodological considerations that need to be taken forward in future research.

## Challenges to recording, categorizing and understanding data

The lack of comprehensive, high-quality data on health and mortality by ethnicity is a significant obstacle to understanding inequalities in health, and therefore how the diverse health needs of different population groups can be addressed. Approximately 90,000 infants are admitted to National Health Service (NHS) neonatal units annually in England, Scotland and Wales. Whilst data may be collected locally, no data are collected at a national level on the number of babies that require a palliative approach to care. This paucity of data presents considerable challenges in informing practice and providing appropriate services for babies and families. It has been estimated that around 2% of the babies admitted to neonatal units in England and Wales have potential palliative care needs.^8^ This number is likely to have underestimated the true need for neonatal palliative care as it is based on neonatal unit admissions and does not consider babies cared for in hospices or the community. As will be stressed throughout this article, it is imperative the different settings in which palliative care may take place are considered.

Available data also show marked disparities in neonatal deaths across population groups. However, it must be remembered that neonatal mortality statistics can only be used as a proxy measure for palliative care need – not all neonatal palliative care will result in death in the neonatal period so these figures may underestimate the true need for neonatal palliative care. Whilst this data are reported regularly, the two main bodies collecting this data [MBRRACE-UK and Office for National Statistics (ONS)] report this using different measures (20+0 weeks gestational age and 24 weeks gestational age, respectively) undermining the ability to meaningfully compare data. ONS data^31^ shows that the overall rates of neonatal deaths have decreased in the United Kingdom in recent years, from 3.2 to 2.7 deaths per 1000 live births between 2007 and 2019, however levels of inequality have remained consistent or have even grown between ethnic groups. For example, there was a disparity of 1.3 deaths per 1000 live births between White British (2.8) and Black Caribbean (4.1) neonates in 2007, compared to a disparity of 3.2 deaths per 1000 live births between White British (2.3) and Black Caribbean (5.5) neonates in 2019.^31^ More important than just documenting differences between groups, however, is to seek an understanding of the underlying mechanisms that cause disparities in the first place.^32^

To be able to use and understand the data in this way, there is a need to critically explore the ways in which data are currently being recorded and categorized (albeit often unintentionally) as non-neutral entities that can reflect both specific power constellations, and unfold particular effects.^33^ As Koffman *et al*.^34^ state that data are ‘created and patterned by the assumptive determinations of researchers to collect items of information and not others, to interrogate some relationships over others, and to see truths in some and not others’. As such, those administering data collection and analysis need to be aware of the potential for and ways in which data may impact on health provider and patient/family perceptions and experiences within neonatal medical settings; a possibility that may be amplified for marginalized groups where physician–patient power relationships may be especially acute.

Across the NHS there are issues in the availability and quality of ethnicity data reducing understandings of inequalities and subsequent opportunities to identify effective responses. The Nuffield Trust argue that current challenges range from the absence of ethnicity data in essential data sources such as death registrations from which mortality statistics are derived, to poor coverage in primary care data, outdated ethnicity codes used within the NHS compared to those used in the most recent census, and systematic differences in ethnicity coding between White and minority ethnic groups.^35^ In addition, accuracy is often low, with 20–35% error in coding of major ethnic minority groups in NHS hospital records when compared to self-reported ethnicity.^36^ Iqbal *et al*. suggest that the reasons for poor quality ethnicity data may include a lack of understanding on the importance of data, reluctance of staff to ask for data, fears over patient reactions and confusion about categorization.^37^ Looking at neonatal care admission in England and Wales specifically, the Neonatal Data Analysis Unit records show how the reporting on maternal ethnicity is decreasing year by year – peaking at 92% in the first year the unit reported on maternal ethnicity in 2013, before dropping by almost 25% in less than 5 years (data were entered for 67.9% of all infants in 2017).^38^ It is worth noting that whilst the Neonatal Data Analysis Unit uses ‘maternal ethnicity’ to categorize infants admitted to neonatal care, ONS and MBBRACE use ‘ethnicity of infant’. The variation of categories from one study to the next therefore makes comparing across studies in any meaningful way very difficult.

Furthermore, migration status is infrequently collected in either neonatal palliative care settings or in health care more generally. For example, differences in primary care utilization between migrants and the UK-born population have relied on self-reported surveys with limited sample sizes.^39^ This lack of detailed, comprehensive and accurate information limits the possibilities for monitoring and improving migrant health, and for conducting comparative studies on inequalities in and access to health care.^40^ Whilst acknowledging that data generated in these categories could feed into stigmatization and ostracizations of im/migrant groups,^33^ collected sensitively and appropriately it can illustrate and address the legitimate needs of these groups to improving services and care. To neglect ethnicity or migration status as a variable in palliative care research may in fact disregard the reality of social stratification, injustices and inequities with implications across the palliative care spectrum of interest.

Alongside this, it is crucial to avoid the use of crude ethnicity labelling, such as ‘South Asian’, as ethnic groups are not homogenous^41^ and such terms fail to account for rich cultural, social and religious nuances that define ethnicity as well as disguising important differences that relate to provision of holistic palliative and bereavement care such as country of origin, religion, language and diet. This lack of precision can also mask inequalities as they are experienced by different ethnic groups and relate to additional needs going unmet. For example, neither the ONS or MBRRACE-UK reports specifically categorize for Traveller communities, with Gypsy Romany, Traveller or Irish Traveller being included as ‘White other’ – however, data from Ireland have shown infants of Travellers to have a mortality rate of 14.1% compared with 3.9% in the non-Traveller population.^42^ It is imperative that this data are collected sensitively – research from Hospice UK into Gypsy and Traveller experiences of palliative care found that a wariness of disclosing Gypsy, Romany or Traveller identity for fear of discrimination meant that staff might not be aware of the additional support that might be required, for example, hospitals and hospices typically rely on written distribution of information which may not be accessible.^43^ Improvements can be made by ensuring that labels are sufficiently granular as to capture important heterogeneity, and that they are employed systematically to decrease confusion around categorization and permit easier data collection and pooling.^41^ Furthermore, in using this data, appropriate attention must be paid to the ways in which the variable of ethnicity intersects with other forms of social difference.^34^ Taking an intersectional approach to data analysis is critical to understanding the underlying mechanisms that cause disparities, rather than just documenting difference.

More robust data would help to illustrate and address the legitimate needs of minority groups for improving services – as well as help identify whose voices are underrepresented. Understanding the barriers and facilitators to collecting this data from healthcare professionals’ perspectives would also inform meaningful recommendations on improved data collection. Initial research from Iqbal *et al*.^44^ suggests that data collection could be improved by allowing time for staff to attend ethnic monitoring training courses to understand how best to collect this information and why it is important to service provision, in turn giving them confidence to explain this importance to the patients in their care.

## Whose voices are missing?

### Families

The majority of published research written in English on neonatal death has been conducted in the United Kingdom, Australia or the United States, and primarily consists of qualitative studies on the experiences of White, heterosexual mothers.^45–49^ Although research has started to emerge in recent years, particularly from Australia,^50–52^ fathers’ experiences are far less studied. Gender stereotypes may compound cultural assumptions, as reported by Bliss^53^ who identified how fathers from South Asian backgrounds found it harder to participate in care when they felt staff assumed that they would not want to be involved. At present, little is known about the experiences of culturally diverse families receiving palliative care or the challenges facing healthcare professionals working in the specialty.^6,28^ This lack of specific research focusing upon ethnically diverse families may in turn deter these families from sharing their experiences.^54^ Furthermore, it is important to consider that the neonatal palliative care journey may not only be experienced by the infant’s parents, but other significant family members such as siblings and grandparents who are frequently underrepresented in research.^55,56^

Culture does not solely equate to ethnic identity, nor does it merely refer to people who share the same racial heritage. Culture can be defined as the ‘shared, overt and covert’ understandings of a group that constitute conventions and practices.^57^ Culture is therefore a dynamic concept, and comprises elements that define, as well as contribute to or detract from, health. In a review of the qualitative literature on palliative and end-of-life care for people from ethnic minority backgrounds, Jones^58^ found that cultural aspects beyond formal religious beliefs were often overlooked. Kodjo^59^ similarly describes how a biomedical model used in most western medicine is often at odds with the cultural context in which people live their lives. Indeed, others have argued that this biomedical model has largely been developed by and for a particular type of patient: ‘the wealthy, white, able-bodied, cissex, endosex and cisgender male’ which not only has ‘real and dangerous implications for those who differ biologically in that they remain underrepresented in health research and the medical professions, but it also has equally real and dangerous implications in the way medicine is socially enacted’ (p.1955).^60^

There has been a considerable effort from the voluntary, community and social enterprise (VCSE) sector to listen to culturally diverse voices in palliative care (e.g. Together for Short Lives and Hospice UK, 2015; Compton Care, 2018; Hospice UK, 2018; St Francis Hospice, 2022).^43,61,62,63^ However, although some reports do look at the experience of neonatal care, neonatal mortality and baby loss (e.g. Sands^64^; Bliss^53^), very few of these reports consider neonatal palliative care specifically, and certain groups are still largely missing from both the published and grey literature. For example, little is known about the experiences of neonatal palliative care for forced migrant families. There is no data available on the numbers of forced migrants with palliative care needs;^28^ however, the combination of trauma related to migration and the complexities of life with a severely ill infant results in multifaceted needs that amplify the need for culturally sensitive care. Despite the current period of ‘super-diversity’,^65^ there is a surprising lack of paediatric and specifically neonatal literature regarding cultural sensitivity in care. This gap merits exploration to better understand the experiences of neonatal palliative care specific to culturally diverse families.

### Multidisciplinary care teams

In addition to gaps in the literature around families, little is known about the challenges faced by the multidisciplinary and interprofessional healthcare team members supporting culturally diverse families during their neonatal palliative care journey. Beckstrand *et al.*^66^ note that whilst families are reliant on nurses more than on any other health professional before and after a death in the neonatal intensive care settings, little is known about the experiences of nurses providing neonatal palliative care. Despite being determined as essential,^67^ the coverage of culturally competent care in the curriculum for nurses and doctors remain sporadic in practice.^68,69^ Furthermore, Calanzani *et al*.^70^ note that cultural competence training for healthcare professionals tends to focus on specific ethnic groups, which fails to consider variations in practice and can lead to assumptions and stereotyping.

Nonclinical professionals such as chaplains also play a part in the multidisciplinary care team, though their role is frequently misunderstood and questioned in light of changing religious and spiritual landscapes.^71,72^ Growing cultural diversity means healthcare providers must care for individuals from a broad range of religious beliefs and some argue that chaplains are one of the few members of a multidisciplinary care team with professional preparation in diverse religious traditions.^73^ Specific concerns around access to hospital religious and spiritual care provision for those of minority faith groups suggest possible deficiencies in the quality of care and support available to patients, carers and staff.^74^ Indeed, a 2004 survey of hospital chaplaincies by Sheikh *et al*.^74^ identified a comparative disadvantage to non-Christians in relation to access to space for worship, chaplaincy staff and quality of chaplaincy care. Despite its vital potential, there is limited empirical evidence related to the role, skills and competencies of healthcare chaplains.^75,76^ Little is known about who is accessing chaplaincy services (e.g. parents, wider family members, healthcare professionals), how (referral or self-referral), when and why. Such research could help guide the development of mechanisms to ensure that such services are competent to cater for, and sensitive to the needs of, diverse populations.

More generally, it can be difficult to measure the impact that nonclinical professionals have on families in receipt of neonatal palliative care. Together for Short Lives report that whilst it is ‘difficult to prove statistically the impact of having cultural link workers, they are a conduit in empowering families to make informed choices and are well placed to reduce stigma and myths within communities’.^77^ Yet no data are collected on cultural link workers, liaison officers, outreach officers, cultural mediators or similar roles that may be present in hospital, hospice or community settings so it is unknown how many families might have access to this resource. Research from 2001 describes such schemes as patchy, disconnected and limited by low awareness of their need, low pay, low status and a lack of professional recognition,^78^ and whilst there is nothing to suggest this will have changed, an updated assessment of nonclinical professionals’ role and impact in the neonatal arena would be welcomed.

In asking ‘whose voices are missing?’ this article has set out to review a wide set of resources to identify gaps in the evidence base. It has identified a lack of evidence on the experiences of ethnically diverse families receiving neonatal palliative care, as well as significant gaps in understanding around the experiences and needs of the multidisciplinary care team.

## Key areas for further research

### Navigating and negotiating environments of care

The evidence base for the lived experience of diverse families accessing, navigating and negotiating challenges in neonatal palliative care settings is scant – largely consisting of grey literature and studies of singular groups. Furthermore, research has tended to treat the different settings in which neonatal palliative care (hospital, hospice, community) in silos, prioritizing the hospital setting, despite the possibility that families may access any combination of environments. There are currently no robust records which capture when a baby moves from tertiary care settings to hospice or community settings. Given the high numbers of babies surviving to discharge^9^ the experiences and perspectives from the community setting should not be ignored.

Children’s hospices are the dominant provider of children’s palliative care in the United Kingdom^61,79,80^; however, there is a paucity of data quantifying the role of hospices in delivering neonatal palliative care. It has previously been reported that 98% of neonatal deaths occur in the hospital, with very small numbers dying at home or in a children’s hospice.^81^ A 2011 survey found that despite 93% of neonatal units surveyed (*n* = 29), having access to hospice support only 63% referred to these services.^81^ Price and Mendizabal-Espinosa^82^ identified complex challenges around hospice staff’s experiences of providing palliative care for infants referred from the hospital, including an ‘us and them’ culture, hospitals holding onto patients due to lack of education about hospice services and staff feeling that parents wanted their babies to be kept in the hospital environment. A more recent survey of UK children’s hospices (*n* = 30) found that the majority did not collect specific data on the number of perinatal referrals, but for the 12 services that did, the majority had seen an increase in referrals over the last 5 years.^83^ Although this suggests that referrals to hospices are increasing, the small sample size makes it difficult to extrapolate the findings and it is possible that families may still be missing out on the holistic palliative care services that hospices can provide.

Traditionally, people from culturally diverse backgrounds have been considered underrepresented in hospice and palliative care provision, although it has not always been clear why this is the case.^84–86^ Although ample research quantifying disparities in hospice use between White and culturally diverse patients has been conducted, there is far less research into the core values of hospice, which began in an Anglo-Christian context and are often still Christian foundations,^87^ and how those values may clash with the end-of-life needs of culturally diverse families and contribute to reduced access to end-of-life care. While the medical community considers issues such as language barriers and patient awareness as possible causes for hospice underuse by culturally diverse patients, it tends to ignore the fact that many parts of hospice working draw on culturally bounded notion of a ‘good death’ that is not necessarily universally appealing. Challenges when caring for culturally diverse families may for example, relate to differing perceptions and understandings surrounding the notion that palliative care implies end-of-life or giving up on active treatment.^88,89^ A lack of focus upon cure by healthcare professionals can thus create further ethical challenges for families to navigate, particularly when counter to their religious beliefs. In-depth qualitative research is thus critical to understand conceptions of a good death and what this means for culturally sensitive palliative care.

The neonatal intensive care unit (NICU) represents a unique environment in its early, intensive and often prolonged treatment of an infant. For many parents, their first experience in an NICU is a profound shock, with their infant being attached to wires, cables and equipment in a place that is far different from what they had planned.^90^ This can be a traumatic experience, with parents reporting being frightened by the technological environment which can make them feel like outsiders and may delay their parental involvement in caregiving.^91,92^ Whilst western cultures may regard parents as central to decision-making in neonatal palliative care settings, in many other cultures people have a more collectivistic view about how healthcare decisions should be made. For example, the Traveller community rely heavily on support from the extended family especially during times of crises and bereavement.^93^ Consideration therefore needs to be given to ensuring the involvement of the extended families in the care of the infant and in the support of the parents, as these will likely be the mainstay of support for the parents once they leave the hospital or hospice environment. Other authors report on how the spatial environment can lead to religious needs going unmet – such as a lack of space for prayer.^94,95^ Researchers need to work with families to understand how they navigate these challenges in the midst of neonatal palliative care and how healthcare professionals can help to negotiate these challenges to ensure a total approach to care.

### Informal support

It is important to also recognize the diversity of noninstitutional settings in which care and support can take place. Yet, at present, little is known about these diverse environments of care and support beyond the institutionalized environments of the hospice and hospital for families receiving neonatal palliative care. Research from Sands suggests that where more formal support networks exist, it may not always be suitable. For example, peer-support groups which are facilitated, and attended, by predominantly White people can feel unsafe or unrelatable.^64^

Informal support networks can play a crucial role during times of crises and grief; However, cultural stereotyping can lead to assumptions about the informal support networks that diverse families utilize – for example, there is a common assumption that minority groups will be from larger families or have community support behind them equating to a greater capacity for, and provision of, informal support.^53,69,96^ This has been challenged in the literature,^95^ with some authors pointing out how acculturation – the process by which minority cultures gradually adopt the values and ethos of the majority culture^97^ – has for many families resulted in the erosion of traditional support networks. Similarly, those families more recently arrived in the United Kingdom, for example, asylum seekers, may not have established support networks. It is vital therefore that research seeks to understand the role of informal support networks in supporting families whilst receiving and following neonatal care. This would include the role played by the VCSE sector, which often goes underrecognized, as well as that of family, friends, faith groups and community.

### Communication

Communication underpins every element of care – in practice, the success of palliative care will primarily be determined by the ability to give and receive information and to respond appropriately.^55^ Effective communication goes beyond language – it includes the ability to understand and empathize with a variety of cultural practices and beliefs.^55^ Poor communication – associated with a lack of sensitivity to cultural and religious issues and lack of translation resources^69^ – can make the provision of neonatal palliative care difficult for parents and professional care teams alike. Furthermore, inequalities in power, which are inherent in the physician–patient encounter, can be exacerbated by communication difficulties, particularly if a patient/family member has a low level of English or is from a different cultural background.^98^

Studies have repeatedly shown provision of translation and interpretation services to be patchy and inappropriate.^99,100^ Findings from the Birthrights inquiry into racial injustice and human rights in maternity care^99^ identified lack of appropriate interpretation and translation services as a systemic issue which prevents women from making informed choices about their or their baby’s care. Often in healthcare settings with limited interpreting resources, interpreters will be saved for ‘essential’ conversations; however, in neonatal palliative care settings conversations will frequently be critical and sensitive. Reviews by Khan^101^ and Rayment-Jones *et al*.^102^ also found evidence of a lack of flexibility and accessibility of interpreting services, especially in urgent situations, which meant that family members had to interpret in their place. This practice is not a recommended norm especially when discussions with the family concern medical prognosis and treatment, symptom control or end-of-life care plans. However, it is noted that some families may prefer to use known and trusted family members, suspicious of the level of confidentiality interpreter services provide.^102^

Families may also become problematized in care settings because of issues with language, leading to the breakdown of communication and care. Mancini and Steele^55^ suggest that parents are acutely aware and in tune with different layers of communication, not just between parents and staff but also among staff. Feelings of isolation and alienation in an unfamiliar setting may be furthered if families perceive staff to be talking about them,^103^ or if families perceive others around them to be receiving different levels of care. A study from the United States^104^ highlighted how medical providers have been shown to demonstrate fewer nonverbal expressions of support and are less likely to offer quality care options to Black patients. This indicates that implicit bias contributes to poorer communication and trust building, critical factors in accessing supportive palliative care,^105^ and that this can lead to a lack of accountability and inadequacy of protection from potential harm to vulnerable patients.

Communication issues can also cause considerable uncertainty for professionals caring for culturally diverse patients .^70^ Inability to communicate effectively with minority ethnic families can cause negative feelings, stress and dissatisfaction among professionals,^78,94^ and nurses distancing themselves from patients.^78^ This may result in hesitancy and inertia around what is best for the patients.^106^ There is limited research which addresses the challenges and effects of communication difficulties from the professional care provider’s perspective.

Research with culturally diverse families exploring what features make for a positive experience with interpreters and how this may differ across family members will be vital to improving interpreter services and culturally sensitive care. This would be complemented by research with interpreters, whose voices are very seldom heard, to understand what they perceive to be the challenges of working to support families experiencing neonatal palliative care.

### Bereavement

Bereavement support is an integral element of neonatal palliative care, which enables families to prepare for and cope with losses.^56^ The literature on parental perspectives highlights the profound loss of ‘normal’ pregnancy and birth and compounded grief caused by minimal acknowledgement from society or medical professionals.^107^ Lundqvist *et al*.^108^ describe the impact of perinatal loss as helplessness and despondency, ‘broken dreams’ and a shattering of connection and self-worth. The complexity of parental perinatal grief has also been linked to complicated or intense grief and associated with a higher risk of developing depression, anxiety and PTSD.^109^ Again, despite a substantial body of literature on women’s support needs following perinatal death, there is limited research that has investigated what supports are best suited for men.^50^ This is especially important as there is emerging evidence around differences in how mothers and fathers accept loss and grieve, and that clinicians aware of these differences may be better able to support families in bereavement. Given the detrimental health and wellbeing outcomes experienced by parents following neonatal loss, it is essential to understand bereavement, and the factors associated with worsened or improved outcomes. Findings from Sands^64^ and Marie Curie^110^ suggest that there is a lack of culturally sensitive bereavement support – particularly for older family members as counsellors can often only speak English and may have received little in the way of training in working with culturally diverse families. It is vital that future research takes an engaged approach to working with bereaved parents, and extended family members, taking an intersectional lens, to understand their support needs following neonatal death.

### Support for nurses

There is an urgent need for qualitative research with nurses (from hospice, community and hospital settings) providing neonatal palliative care to culturally diverse families to understand the barriers and enablers they face in care provision, their educational and training needs, as well as their own wellbeing needs.

This review has highlighted a need for particular attention to two areas: bereavement and spiritual care. Evidence suggests that whilst bereavement support is considered an inherent feature of nursing practice across intensive care settings, not all nurses feel educationally prepared for this role. Nurses surveyed reported feeling less confident in providing culturally sensitive bereavement support (an average confidence rating of 2.56 compared to 3.2 for their role generally).^111^ Similarly, nurses are increasingly encouraged to provide spiritual support to patients.^112–114^ However, they often receive little or no formal training in this area,^115^ and there is limited up-to-date guidance. The RCN *Spirituality in Nursing Care: a Pocket Guide* dates back to 2011 and The Nursing and Midwifery Council Code (2015, updated 2018) makes no reference to spiritual care despite this expectation upon nurses.^112,116^ As such, nurses report lacking confidence in providing this aspect of care.^117^

### Methodological considerations

In shaping a research agenda, we must be mindful that the ways that we conduct research may exacerbate the gaps which we are trying to fill. There has been a tendency across disciplines to deem ethnically diverse communities as ‘hard to reach’ – as such focusing the attention upon the research participants themselves, rather than the paucity of research where researchers are sufficiently embedded in the community to build trusting relationships. Hussain, Koffman and Bajwah argue that this reflects an ‘assimilation perspective where certain racial and/or ethnic groups are considered to be deficient, lacking, or inferior in their behaviours [. . .] underlying this, perhaps well intentioned, approach is the assumption that one group, usually the dominant culture, reflects the gold standard’.^118^

How culture may influence and determine the holistic care of families receiving neonatal palliative care cannot be ignored. Taking an engaged approach to research^119^ is vital to understand the distinct experiences of diverse groups based on what they see as their needs and priorities. The purpose of such research is not to reduce cultural aspects of palliative care to an interpretive list of end-of-life beliefs and practices for specified groups, creating a ‘fact-file’ or ‘checklist’,^120^ but to ensure culturally sensitive care. Public involvement and engagement must sit at the heart of future research, with advisory boards formed from those with lived experience. Research must be with and for, not merely about, those voices we have identified as currently missing from the literature.

## Recommendations for research

- Key areas for further research with culturally diverse families identified throughout this review (with all research including engagement by design):
  - inclusion of the lived experiences of families to inform palliative care systems that enable equity in neonatal palliative care settings;
  - understanding conceptions of what constitutes high-quality palliative care from diverse perspectives;
  - understanding what constitutes effective and empathic communication between families and professionals and how this can best be enabled;
  - how best to provide culturally sensitive forms of bereavement care for culturally diverse families.
- There is also an urgent need for research with members of the multidisciplinary care teams to understand the barriers and enablers they face in care provision and multisectoral working; their educational and training needs; and what is required to support their own wellbeing needs.
- Alongside this, we urge that health systems managers and practitioners reflect on what is needed to support a whole systems approach to more sensitively support culturally diverse families within neonatal palliative care settings (see e.g. WHO^121^ which considers questions for healthcare practice at systemic, organizational, professional and leadership levels).

## Conclusion

This article has sought to review and set out key questions that need to be asked to deepen the understanding of neonatal palliative care for culturally diverse families. In bringing together a wide range of resources from both academic and grey literature, we have identified gaps in knowledge and in the empirical evidence base.

In critically reviewing the categorization and recording of ethnicity data collection, this article has called for improvements to address the significant gaps in the data that would allow for a true picture of the health disparities in neonatal palliative care need and mortality. This article has also recognized that we must go beyond simply documenting difference – in calling for an intersectional analysis of this data we seek to understand the underlying mechanisms causing these disparities.

As the article has highlighted throughout, there is a lack of research that listens to those from culturally diverse families with experience of neonatal palliative care. In asking whose voices are missing it is apparent that it is not just the family perspectives that are missing from the literature, but also clinical and nonclinical members of the multidisciplinary care team within and beyond formal healthcare settings. Listening to these voices will allow for a deeper understanding of the inequities in service provision as well as identify priorities for improvement.

Key areas for further research have been identified, including: the lived experiences of families and how they can navigate and negotiate challenges in neonatal palliative care settings; conceptions of a good death from diverse perspectives; effective communication between families and professionals; and the need to provide culturally competent forms of bereavement care for culturally diverse families.

Finally, the article has called for an engaged approach to future research. We argue that it is vital that future research into neonatal palliative care seeks a better understanding of the ways in which culture influences experiences of care, and we have emphasized the need for listening to and centring the voices of culturally diverse families to understand their distinct and unique experiences.

## Declarations

**Ethics approval and consent to participate:** Not applicable.

**Consent for publication:** Not applicable.

**Availability of data and materials:** Not applicable.
